# Commentary on “Evidence of complement dysregulation in outer retina of Stargardt disease donor eyes”

**DOI:** 10.1016/j.redox.2021.101957

**Published:** 2021-03-24

**Authors:** Patty P.A. Dhooge, Timo W.F. Mulders, Carel C.B. Hoyng

**Affiliations:** Department of Ophthalmology, Radboud University Medical Centre, Nijmegen, the Netherlands

**Keywords:** Stargardt, ABCA4, PRPH2

We read with interest the article entitled “Evidence of complement dysregulation in outer retina of Stargardt disease donor eyes” by Jane Hu et al. published in *Redox Biology* (https://doi.org/10.1013/j.redox.2020.101787)*.* We want to congratulate the authors for presenting the first direct evidence of RPE complement dysregulation as a causative factor in developing Stargardt phenotype. However, a note of caution is due here since Stargardt disease (STGD), defined as a disease caused by mutations in the *ABCA4* gene, was genetically confirmed in only one out of three included donor eyes.

Donor eye #3 harbors a *PRPH2* mutation without a *ABCA4* mutation and can therefore not be diagnosed as STGD. It can be challenging to distinguish STGD from *PRPH2-*accosiated disease, as the type and distribution of flecks caused by *PRPH2* variants strongly resemble the fundus flavimaculatus in STGD1 [1,2]. In fact, 5–10% of patients with a phenotype resembling STGD but without variants in *ABCA4* after complete locus screening, had variants in *PRPH2* [3]. Even though the Stargardt diagnosis of donor eye #3 was argued in the discussion section, Jane Hu et al. fail to acknowledge the significance of the genetic *PRPH2* diagnosis and stick to initial clinical diagnosis that has overlooked the *PRPH2* phenocopie of *ABCA4* disease. We therefore advocate the use of the term pseudo-Stargardt or autosomal dominant multifocal pattern dystrophy simulating STGD1/fundus flavimaculatus for donor eye #3.

The study by Hu et al. included donor eyes clinically diagnosed as STGD1. However, because of the clinical heterogeneity within STGD1, ranging from an early age of onset with a cone-rod phenotype in progressed disease, to a late disease onset with foveal sparing [4,5], patients present with a range of phenotypes at consultation to an ophthalmologists. Because of these conditions it may happen that patients get several diagnoses because of different stages of disease during life. We therefore underline the importance of proper genetic screening to diagnose STGD1. Unfortunately, with the already fixed tissue, retrospective genetic data did only provide evidence for *ABCA4* disease in donor eye #1. Evidence for complement dysregulation in STGD/*ABCA4* disease cannot be established if one only includes a single eye with proven *ABCA4* mutations. This is an important issue for future research.

In addition, it is important to establish the correct genetic diagnosis in preclinical research as clinical trials only include patients with a specific genetic profile. Patients are currently being treated in a phase 2b clinical trial with a complement inhibitor [NCT03364153], but only if they harbor at least two pathogenic mutations of the *ABCA4* gene. However, according to the data of Jane Hu et al. patients with *PRPH2* mutations might also benefit from complement inhibiting therapies. Further studies that look into the role of the complement system within pseudo-Stargardt patients will need to be undertaken.

## Declaration of competing interest

No conflict of interest.
